# Limb-Shaking Transient Ischemic Attacks Masquerading as Focal Seizures

**DOI:** 10.7759/cureus.8157

**Published:** 2020-05-16

**Authors:** Abdullah Alkutbi, Ahmed Elkady

**Affiliations:** 1 Neurology Department, International Medical Centre, Jeddah, SAU; 2 Neurology Department, Saudi German Hospital, Jeddah, SAU

**Keywords:** transient ischemic attacks, limb shaking, carotid stenosis, hypoperfusion, focal seizures.

## Abstract

Limb shaking is a paroxysmal involuntary hyperkinetic movement that may be a presentation of severe unilateral steno-occlusive carotid disease. This unusual form of transient ischemic attack (TIA) is often misdiagnosed as focal motor seizures, especially with frequent repetition. We present a case of 67-year-old man with severe unilateral carotid stenosis leading to frequent left arm shaking TIAs. Initial work-up did not reveal any abnormalities, and anticonvulsant was started. He readmitted again after few days with left side mild hemiparesis. Cerebrovascular evaluation showed recent watershed infarction with significant stenosis in the ipsilateral internal carotid artery (ICA). The patient underwent stenting of the right ICA with weakness improvement and no more limb-shaking TIA on follow-up. In conclusion, early recognition of limb-shaking TIAs and differentiating it from focal motor seizures can facilitate identification of pre-occlusive carotid stenosis, allowing for appropriate interventions to prevent further TIAs or disabling stroke.

## Introduction

Transient ischemic attacks (TIA) are typically present with various neurological symptoms, including motor, sensory, speech, or visual deficit attributed to perfusion insufficiency. They usually last less than 24 hours, mostly not more than one hour, happening either once or recurrently in high-risk patients [1]. Unilateral involuntary hyperkinetic movements of the leg or arm are rarely reported as a feature of ischemic attack. Moreover, such attacks are often misdiagnosed as focal motor seizures due to its short period and recurrent incidence [2]. The diagnostic difficulty is not only due to their phenomenological similarity with seizures, but also due to the fact that ischemic infarction is the most common cause of focal epilepsy in the elderly [3]. Limb-shaking TIA as a presenting symptom of cerebral vascular disorder has been rarely reported in literature and believed to be associated severe internal carotid steno-occlusive disease [4]. Although the differential diagnosis of limb-shaking movements may be challenging, the prompt recognition of limb-shaking TIA is vital for preventing further attacks or stroke through improvement of cerebral perfusion [5]. Moreover, both extracranial and intracranial stenoses are independent risk factors for stroke after TIA, and early implementation of medical and surgical therapeutic strategies may be crucial to avert further massive stroke [6]. We report a case of limb-shaking TIA being first treated as focal seizure, before a high-grade stenosis of contralateral internal carotid artery (ICA) was identified as the cause of these intermittent movements.

## Case presentation

A 67-year-old man was known to be a heavy smoker; he had also been uncontrolled hypertensive and hyperlipidemic for years. He presented to our emergency department with attacks of rhythmic arm shaking. He reported acutely feeling dizzy and then his left arm started to shake at a rate of about 3-4 Hz. This attack recurred four times lasting for about two minutes each, yet aborted spontaneously without medications. Consciousness was never impaired and there were no other symptoms, except that both frequency and duration increased upon position changing. Moreover, he reported previous similar attacks two months ago, and at that time they lasted only for few seconds. His vitals upon presentation were within normal: heart rate was 78 beats per minute, blood glucose level was 127 mg/dL, oxygen saturation was 96% in breathing room air, and body temperature was 37.5°C. His blood pressure was 130/80 mmHg. Urgent CT of the brain done and was normal. His initial laboratory investigations were within normal ranges, so focal motor seizures were thought to be the most likely diagnosis. He was given a loading of 1,000 mg intravenous phenytoin, but this did not reduce the frequency of the attacks. He was admitted to hospital for extensive work-up including full laboratory investigations, echocardiography, and pelviabdominal ultrasonography, which did not reveal any abnormalities. MRI with gadolinium enhancement administration showed only periventricular ischemic changes and atrophic changes related to age, without any gadolinium-enhanced lesions. Moreover, his epilepsy panel and tumor markers were negative, and prolonged electroencephalogram (EEG) video monitoring failed to capture any epileptic activity, and only continuous slowing over right frontoparietal lobes could be detected. We started him on carbamazepine with a target dose of 400 mg twice daily, along with modification of his antihypertensive medications. Mild improvement of attack frequency and duration was achieved. Meanwhile, the patient was discharged with a plan to follow-up in neurology clinic with recommendation for prolonged cardiac Holter monitoring.

**Figure 1:**
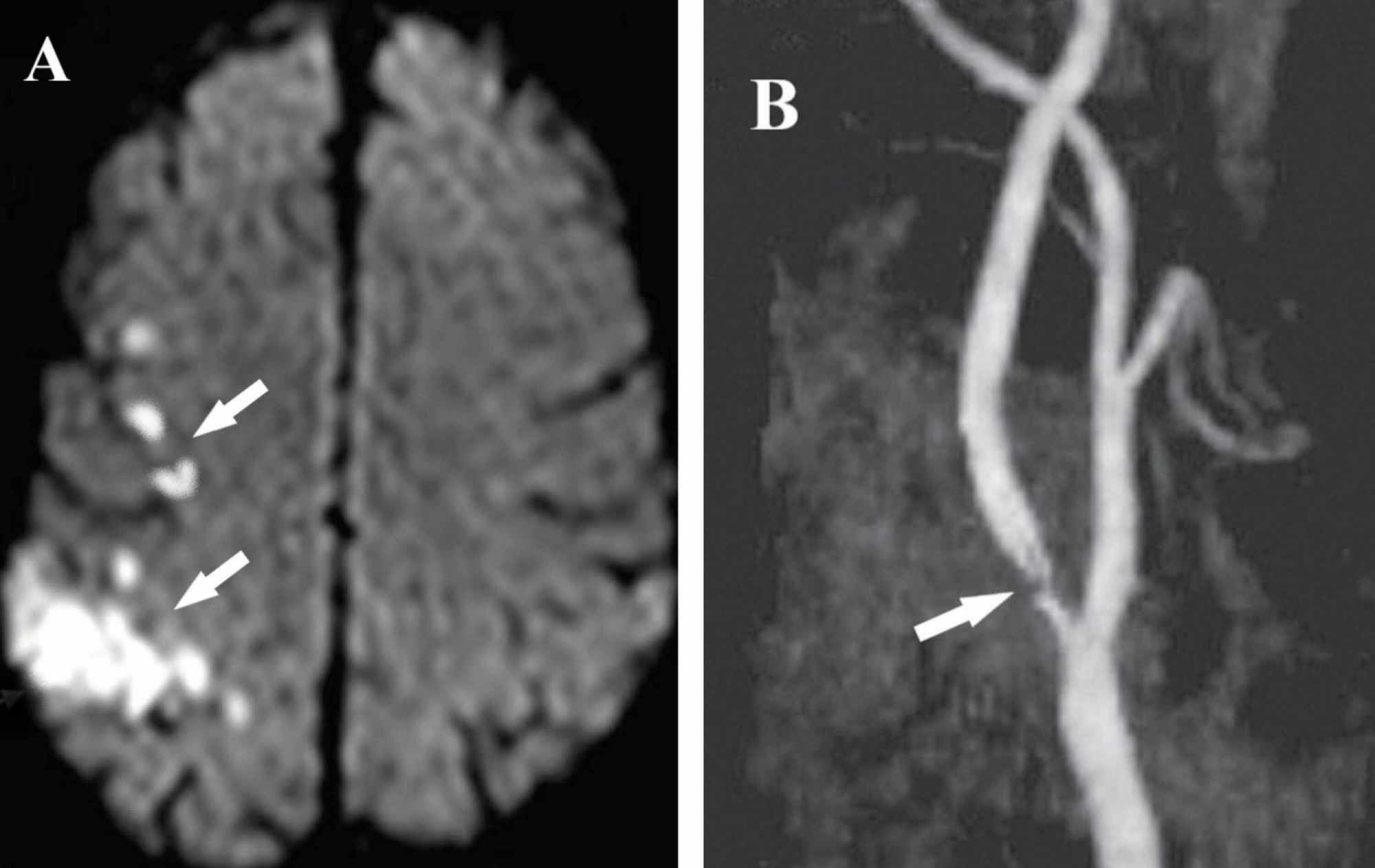
(A) Axial MRI diffusion sequence reveals multiple watershed ischemic lesions (white arrows) involving the frontoparietal cortex. (B) Magnetic resonance angiography of right internal carotid artery showing severe proximal stenosis at its origin (white arrow).

The patient continued to have similar attacks and attended neurology clinic after two weeks with mild left side weakness, upper limb grade 3/5, and lower limb grade 4/5. Although we considered this as Todd's paralysis, new MRI showed watershed infarction with diffusion restriction over right hemisphere (Figure 1A) and so he was readmitted again. Moreover, Doppler ultrasound and magnetic resonance angiography (MRA) of the cerebral vessels disclosed severe stenosis (>90%) of the right ICA at the level of the bifurcation (Figure 1B).

**Figure 2:**
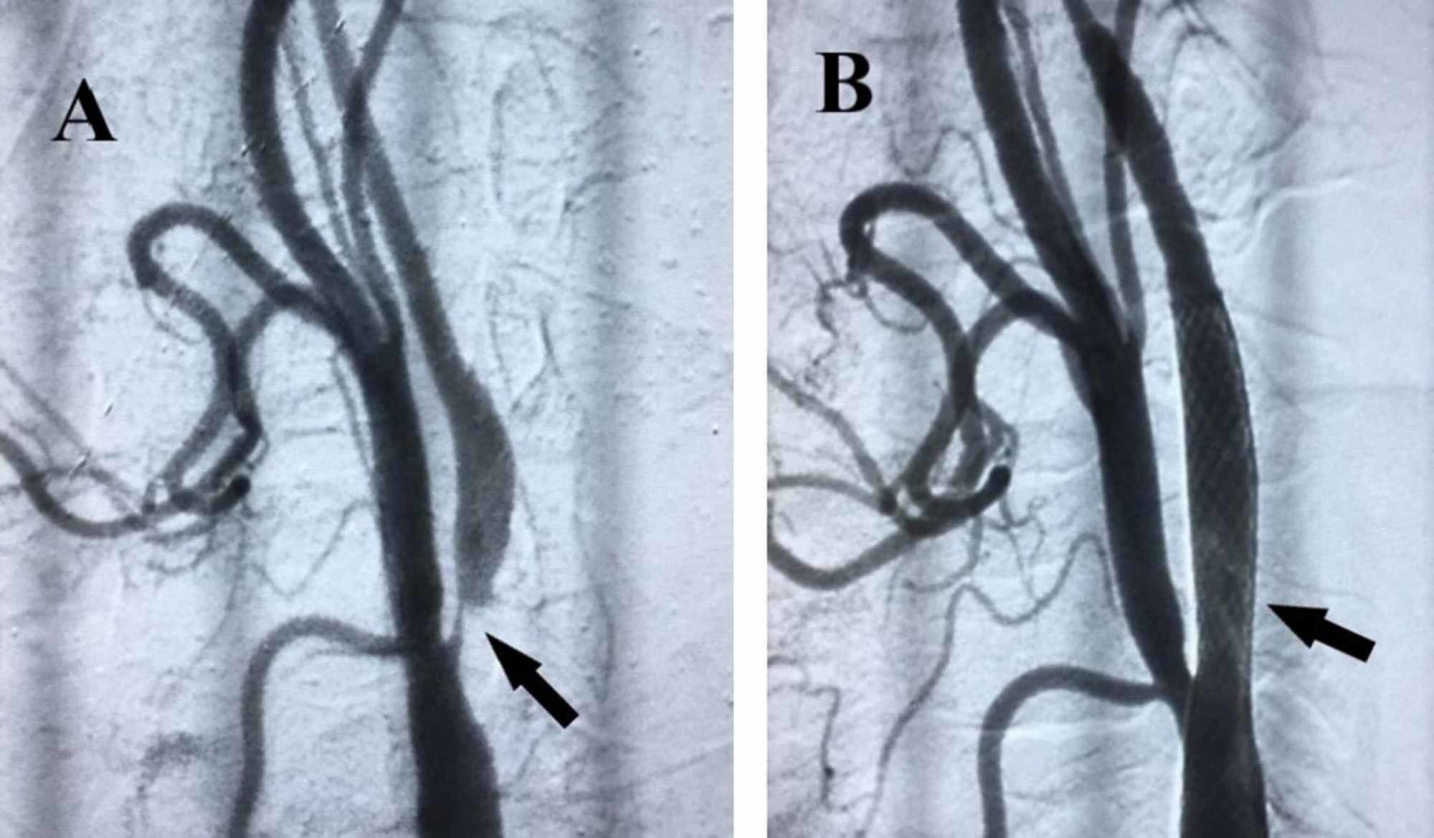
Angiographies of right internal carotid artery. (A) There was a severe stenosis (95%) before stenting (black arrow). (B) After carotid stenting, the stenosis was rectified to a satisfying degree (black arrow).

At this point, a diagnosis of low-flow TIAs presenting with limb shaking, as a result of cerebral hypoperfusion, was made; meanwhile, the patient received intensive medical therapy including dual antiplatelets with high-dose statins and we started to withdraw antiepileptic medications. Furthermore, he was referred to our neurology interventionist for urgent stenting of his symptomatic right ICA, in order to prevent extensive brain ischemia or total ICA occlusion. The procedure was safely done within few days with only moderate headache post stenting (Figure 2), and significant weakness improvement was achieved with physiotherapy over few weeks and the patient remained asymptomatic in the 12-month follow-up period.

## Discussion

Limb-shaking TIA is an infrequent manifestation of cerebral hypoperfusion associated with contralateral carotid stenosis, which was first reported by Miller‑Fisher in 1962 [7]. Although many cases have been well described during last two decades, clinicians often have mistaken them for focal seizures, as in our patient [5]. TIAs, like ischemic stroke, are traditionally believed to be associated with negative neurological symptoms; therefore, a diagnosis of TIA is usually not considered in patients presenting with positive symptoms like paroxysmal abnormal movements [8]. As EEG did not show any epileptic activity in our patient, with normal structural imaging studies, and failure of anticonvulsants to control these attacks, reevaluation of attacks phenomenology with another diagnosis should be considered. Furthermore, the absence of Jacksonian march or extension to face is not a typical presentation of focal seizures [2]. The exact underlying mechanism of the limb-shaking movements is unclear; however, their occurrence in the context of contralateral severe carotid stenosis is speculated to transient focal hemodynamic failure (low-flow TIA) [9]. Although these movements are often precipitated by postural changes and sometimes disappeared after lying down as in our patient, extensive research did not show any decrease of cerebral perfusion with standing or posture changes [10]. New MRI of our patient showed recent watershed infarction within dorsofrontal and rolandic cortices contralateral to the shaking limb. A previous review of limb-shaking patients revealed that 12% of ischemic events within watershed cerebral infarctions, mostly with severe large vessel steno-occlusion, found to experience focal limb shaking [11]. Differentiation of limb-shaking movement from other paroxysmal hyperkinesia like orthostatic myoclonus or primary orthostatic tremor is very devastating, with the latter manifests as involuntary leg movements either shaking or jerking [12].

Tiseo and his colleagues proposed a diagnostic assessment with a stepwise approach for the early diagnosis of limb-shaking TIA. This approach includes proper medical history assessment and better evaluation of the involuntary movements with detailed neurovascular examination. Furthermore, brain imaging should not be limited to traditional neuroimaging, but functional evaluating of cerebral blood flow or metabolism is worth looking for, with exclusion of seizures with EEG [8]. 

## Conclusions

This case report highlights that limb-shaking movement is a rather uncommon form of hemodynamic TIA that should be recognized and differentiated from conditions like focal motor seizures. Moreover, a quick diagnosis by careful neurovascular physical examination along with an accurate medical history is important to abolish the attacks and also to reduce the risk of major stroke.
